# Nanoplastic Disrupts Intestinal Homeostasis in Immature Rats by Altering the Metabolite Profile and Gene Expression

**DOI:** 10.3390/ijms26157207

**Published:** 2025-07-25

**Authors:** Justyna Augustyniak, Beata Toczylowska, Beata Dąbrowska-Bouta, Kamil Adamiak, Grzegorz Sulkowski, Elzbieta Zieminska, Lidia Struzynska

**Affiliations:** 1 Nalecz Institute of Biocybernetics and Biomedical Engineering, Polish Academy of Sciences, Ks. Trojdena 4 st., 02-109 Warsaw, Poland or jaugustyniak@imdik.pan.pl (J.A.); beata.toczylowska@ibib.waw.pl (B.T.); 2 Department of Neurochemistry, Mossakowski Medical Research Institute, Polish Academy of Sciences, 5 Pawinskiego st., 02-106 Warsaw, Poland; 3 Laboratory of Pathoneurochemistry, Department of Neurochemistry, Mossakowski Medical Research Institute, Polish Academy of Sciences, 5 Pawinskiego st., 02-106 Warsaw, Poland; bbouta@imdik.pan.pl (B.D.-B.); kadamiak@imdik.pan.pl (K.A.); gsulkowski@imdik.pan.pl (G.S.); lidkas@imdik.pan.pl (L.S.); 4 Doctoral School of Translational Medicine, Centre of Postgraduate Medical Education, 99/103 Marymoncka st., 01-813 Warsaw, Poland

**Keywords:** polystyrene nanoparticles, jejunum, metabolomics, gene expression, intestinal health, oxidative stress, inflammation

## Abstract

Plastic pollution has recently become a serious environmental problem, since the continuous increase in plastic production and use has generated enormous amounts of plastic waste that decomposes to form micro- and nanoparticles (MPs/NPs). Recent evidence suggests that nanoplastics may be potent toxins because they are able to freely cross biological barriers, posing health risks, particularly to developing organisms. Therefore, the aim of the current study was to investigate the toxic potential of polystyrene nanoparticles (PS-NPs) on the jejunum of immature rats. Two-week-old animals were orally exposed to environmentally relevant dose of small PS-NPs (1 mg/kg b.w.; 25 nm) for 3 weeks. We detected a significant accumulation of PS-NPs in the epithelium and subepithelial layer of the intestine, which resulted in significant changes in the expression of genes related to gut barrier integrity, nutrient absorption, and endocrine function. Moreover, increased expression of proinflammatory cytokines was observed together with decreased antioxidant capacity and increased markers of oxidative damage to proteins. Additionally, in the jejunal extracts of exposed rats, we also noted changes in the metabolite profile, mainly amino acids involved in molecular pathways related to cellular energy, inflammation, the intestinal barrier, and protein synthesis, which were consistent with the observed molecular markers of inflammation and oxidative stress. Taken together, the results of the metabolomic, molecular, and biochemical analyses indicate that prolonged exposure to PS-NPs may disrupt the proper function of the intestine of developing organisms.

## 1. Introduction

With the increasing development of industry and the pace of life, people are constantly exposed to long-term and unpredictable negative factors related to the environment, diet, and lifestyle. All these factors can affect the human body, leading to the induction of chronic unpredictable mild stress, which manifests itself, among other conditions, in digestive disorders, immune dysfunction, and neuromodulatory imbalance, posing many threats to human health. In recent years, interest in the potential role of the intestinal barrier in stress-related disorders has increased [1], as has the involvement of various environmental stressors in this mechanism, one of which is the consumption of an increasing amount of plastic micro- and nanoparticles from food and water packaging [2]. The ubiquity of plastic particles (PPs) in food and beverages results in chronic exposure and poses potential gastrointestinal health risks. Exposure to PPs is currently associated with various inflammation-related intestinal diseases. Among others, the fecal concentrations of PPs of various sizes are significantly greater in patients with inflammatory bowel disease than in healthy individuals [3], which suggests that PPs may be a causative factor of inflammation. Accumulating evidence also supports the intestinal toxicity of ingested PPs and their possible role in the induction of colorectal cancer [4]. Oral exposure to PPs can damage the intestinal epithelium and promote the development of chronic tissue inflammation, thereby increasing the risk of cancer [5]. In addition to inflammation, other toxic mechanisms of PPs in the intestine may also be considered. For example, PPs have been shown to exert immunotoxic effects by inducing dysfunctions of the intestinal immune system [6]. Moreover, experimental data from animal studies have confirmed that both short- and long-term exposure to PPs affects the gut microbiota, causing modifications in microbial communities, thus leading to dysbiosis or imbalance between good and bad bacterial species and consequently to impaired intestinal barrier function [7,8]. This is important in light of the knowledge linking gut dysbiosis to many disorders, such as obesity, diabetes, and hypertension [9], as well as central nervous system (CNS) disorders associated with the dysfunctional gut–brain axis, which is an essential communication network between the gut and the brain [10]. Therefore, the toxic effects of PPs on the intestine may be multifaceted and have negative consequences for the health of the entire organism.

Considering that environmental plastic pollution continues to increase and that health implications have reached the public focus, we need more data about the possible harmful effects of ingested PPs on intestinal function. Therefore, the aim of the present study was to determine whether exposure of immature rats to polystyrene nanoparticles (PS-NPs) influences the small intestine at the cellular level. For this purpose, we checked the profile of metabolites, small (1.5 kDa) hydrophilic and hydrophobic compounds in the jejunum of rats, on the basis of NMR spectroscopy data obtained from tissue extracts. Metabolic profile changes in exposed animals were combined with analysis of the expression of selected genes involved in the proper function of the intestine, as well as genes related to oxidative stress and inflammation, and complemented by biochemical and ultrastructural analyses.

In this study, we used prolonged (3-week) exposure to a low dose of PS-NPs (1 mg/kg b.w./day) via the oral route, which is relatively close to the predicted levels of nanoplastics under environmental conditions and was calculated based on the available literature data [11,12]. We focused on immature organisms, since they are much more vulnerable to toxic insults than adults are, including the toxic impact of nanoparticles [13], and there are no data yet on the developmental toxicity of nanoplastics in animal models. Animals are exposed via the oral route, which is the most frequent route by which immature organisms are exposed to nanoparticle-containing products.

## 2. Results

### 2.1. Accumulation of PS-NPs in the Jejunum of Exposed Rats

Commercially available 25 nm PS-NPs functionalized with an amine group (-NH2) and conjugated with a red fluorochrome with an Ex/Em spectrum of 545/566 nm were used in the study to visualize their presence in the tissue by confocal microscopy. The detailed characteristics of the PS-NPs purchased from the manufacturer are described in our previous papers [14,15]. TEM and zeta potential analyses confirmed that the PS-NPs are spherical in shape, stable in solution and do not agglomerate under conditions close to physiological conditions (37 °C, pH = 7.4).

**Figure 1:**
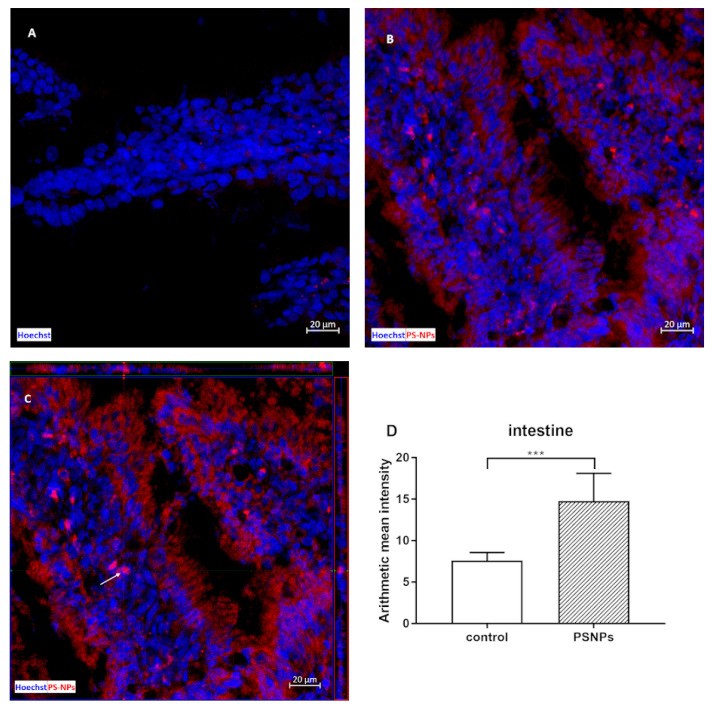
Representative confocal microscopy images of the small intestine obtained from control and PS-NPs-exposed immature rats (PS-NPs conjugated with red fluorochrome). In the control image (**A**), low autofluorescence is observed as scarce red dots, which are limited to small areas of the tissue. In images from exposed animals, PS-NPs-derived red fluorescence is visible as red light dispersed throughout the tissue, staining the epithelial cells and some subepithelial cells (**B**,**C**), Z-stack image of stained cells (**C**) (the arrow indicates the example cells). Hoechst was used to visualize the nuclei (blue). The scale bars indicate 20 µm. Graph (**D**) shows the mean ± SD of red fluorescence intensity in the ROIs defined in 6 jejunal samples collected from control and PS-NP-treated animals; *** *p* < 0.001 vs. control (Student’s *t* test).

The first objective of this study was to determine whether and to what extent small PS-NPs accumulate in the intestines of immature rats after prolonged exposure. We confirmed that 3 weeks of exposure of immature rats to a low dose of PS-NPs resulted in their significant accumulation in the intestine, as revealed by the mapping of PS-NPs-derived fluorescence ex vivo in the isolated tissue (Figure 1). The intestinal tissue from the control rats also presented low red fluorescence, which we assumed was due to background autofluorescence originating from the chemical-rich internal environment. However, the pattern of fluorescence differed according to the source of origin. In control tissue, the intensity of red autofluorescence was low and was visible in the form of scarce red dots of different sizes (Figure 1A). In the tissue obtained from exposed animals, PS-NPs-derived fluorescence revealed a uniform appearance visible on the surface and inside the cells of the epithelial layer, as well as in the deeper subepithelial layer (Figure 1B). The additional use of the 3D structural precision of Z-stack imaging allowed the visualization of red-labeled PS-NPs inside the cytoplasm of distinct cells (Figure 1C), which indicates the ability of PS-NPs to be internalized. The measurements and statistical analysis of the intensity of the fluorescence signals derived from similar regions of interest (ROIs) revealed significant differences between the control and PS-NPs-exposed groups (Figure 1D).

### 2.2. Metabolomics of the PS-NP-Induced Jejunum of Immature Rats

Metabolomics can be defined as the systematic study of specific chemical markers that are produced during various reactions occurring in the tissue. In our study, six samples from the control group and five samples from the exposed group, respectively, were subjected to NMR analysis for hydrophilic compounds. Two samples, one from each group, were removed from the analyses for technical reasons. In turn, seven samples from the control group and six samples from the exposed group were collected for analysis of hydrophobic compounds. The amounts of metabolites are expressed as the magnitudes of their NMR signals normalized to the wet weight of the sample, which corresponds to the concentration of the individual compounds. For the statistical analyses, 45 NMR signals representing different hydrophilic compounds and 26 NMR signals representing different hydrophobic compounds/functional groups were used. The results of MVA, together with the variable importance in the projection (VIP) values, are presented in Table 1 and Table 2, respectively. Variables with a VIP score of ≥1 were considered important.

**Figure 2:**
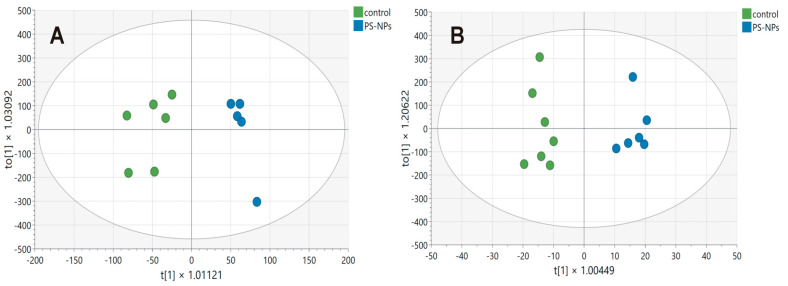
Score plot of the OPLS-DA model for hydrophilic (**A**) and hydrophobic (**B**) compounds detected in the jejunum samples obtained from control (green circles) and PS-NPs-exposed (blue circles) immature rats. The first predictive component, t[1], on the x-axis represents the between-class variation in the predictive component. The first orthogonal component, to[1], on the y-axis represents within-class variation in the first orthogonal component. The ellipses represent the Hotelling T2 with a 95% confidence interval.

In the case of hydrophilic metabolites, MVA allows us to construct a model consisting of three components, one predictive component and two orthogonal components, which fit the data well (R2 = 0.91) and are good for prediction (Q = 0.75). All samples were correctly assigned to their groups (Fisher test, *p* = 0.0022). In turn, MVA of hydrophobic metabolites allows the construction of a model consisting of six components, one predictive and five orthogonal. The model fits the data well (R2 = 0.93) and is good for prediction (Q = 0.42). All samples were correctly assigned to their groups (Fisher test, *p* = 0.0006). The score plots for the model are presented in Figure 2A and Figure 2B, respectively. MVA indicated that the profiles of both hydrophilic and hydrophobic compounds differed between the studied groups. Metabolites significantly influencing the division of groups (VIP > 1) can be indicated for profiles of both hydrophilic and hydrophobic metabolites.

### 2.3. PS-NPs-Induced Alterations in the Expression of Selected Genes Linked to Jejunum Functions

**Figure 3:**
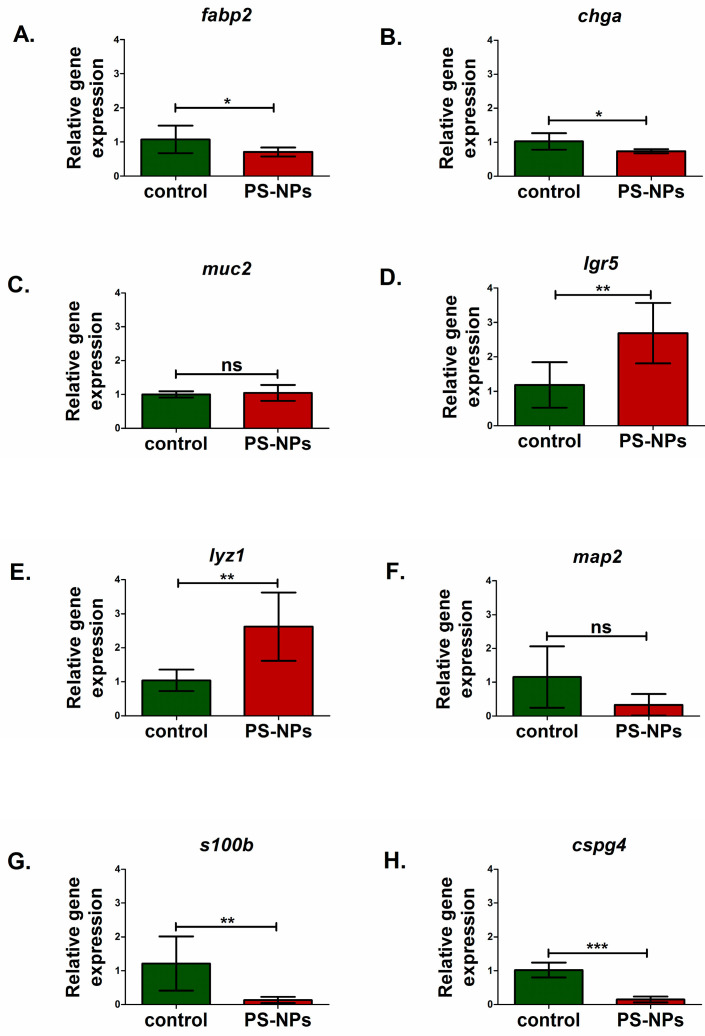
Relative expression of genes encoding selected markers of intestinal cells, including the enteric nervous system cells, in the jejunum of control and PS-NPs—exposed immature rats: (**A**) *fabp2* (enterocytes); (**B**) *chga* (eneroendocrine cells); (**C**) *muc2* (goblet cells); (**D**) *lgr5* (intestine stem cells); (**E**) *lyz1* (paneth, cells); enteric nervous system: (**F**) *map2* (enteric neurons); (**G**) *s100b* (enteric astrocytes); (**H**) *cspg4* (enteric oligodendrocytes). Reference gene validation with the comprehensive gene stability analysis method has been generated with the RefFinder web tool—Figure 4G. The data are presented as the means ± SDs from n = eight distinct experiments. Statistical significance was assessed as follows: ns—nonsignificant, * *p* < 0.05; ** *p* < 0.001; *** *p* < 0.0001 (statistical tests as described in the Section 4).

**Figure 4:**
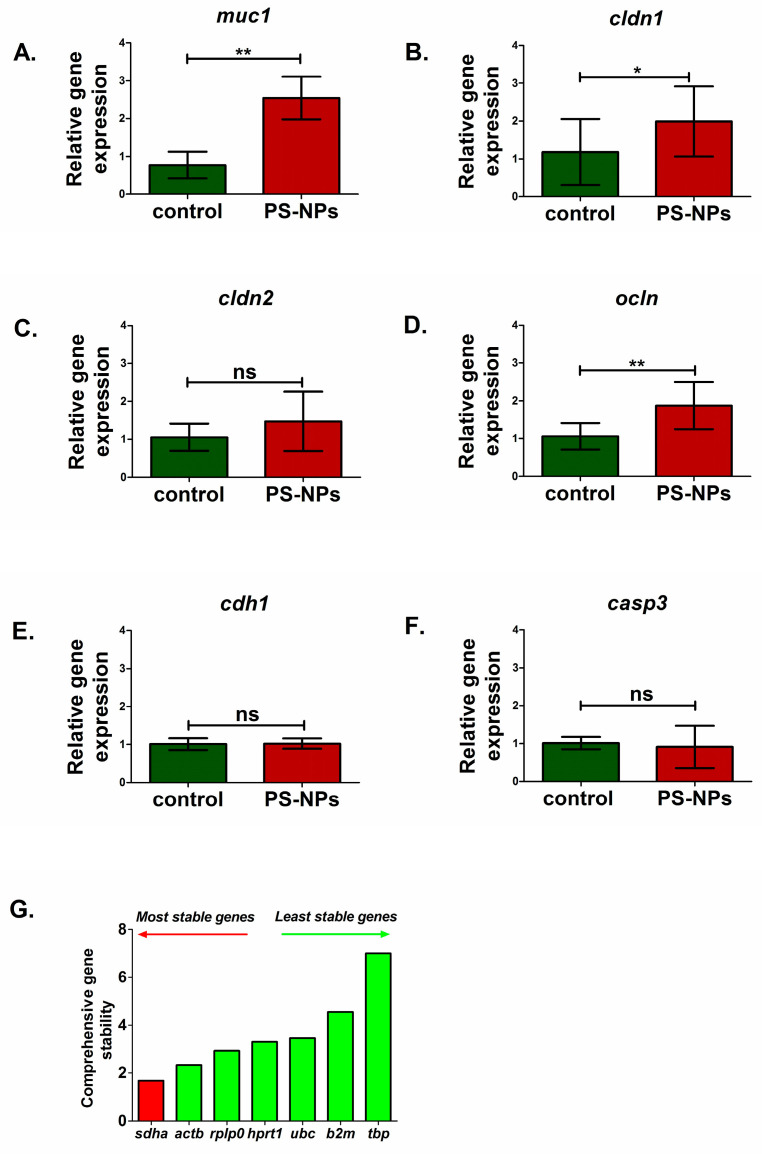
Relative expression of genes encoding selected markers of mucus layer, intestinal barrier and apoptosis in the jejunum of control and PS-NPs-exposed immature rats: (**A**) *muc1* (mucus layer); (**B**) *cldn1* (intestinal barrier), (**C**) *cldn2* (intestinal barrier), (**D**) *ocln* (intestinal barrier), (**E**) *cdh1* (intestinal barrier), and (**F**) *casp3* (apoptosis). Reference gene validation with the comprehensive gene stability analysis method has been generated with the RefFinder web tool (**G**). The data are presented as the means ± SDs from n = eight distinct experiments. Statistical significance was assessed as follows: ns—nonsignificant, * *p* < 0.05; ** *p* < 0.001 (statistical tests as described in the Section 4).

qRT–PCR gene expression analysis of the immature rats’ jejunum exposed to PS-NPs revealed significant changes in the relative expression of several genes crucial for maintaining the main intestinal functions (Figure 3 and Figure 4).

We analyzed markers of the main types of cells present in the jejunum, such as enterocytes (*fab2*), enteroendocrine cells (*chga*), intestinal stem cells (*lgr5*), Paneth cells (*lyz1*), and goblet cells (*muc2*). Significant downregulation of the fatty acid binding protein 2 (*fabp2*) gene (*p* < 0.05) and chromogranin A (*chga*) gene (*p* < 0.05) expression was noted in PS-NPs-treated rats (Figure 3A,B) compared with the control, which may suggest disturbances in the functions of enterocytes and enteroendocrine cell types, respectively. In turn, genes such as leucine-rich repeat containing G protein-coupled receptor 5 (*lgr5*) (*p* < 0.01) and lysozyme 1 (*lyz1*) (*p* < 0.01) expression were significantly upregulated compared with those in the control under conditions of PS-NPs exposure (Figure 3D,E). We did not observe a significant effect of PS-NPs on the expression of mucin 2 (*muc2*) specifically secreted by goblet cells within the intestinal lining (*p* = 0.637) (Figure 3C). However, we noted a statistically significant increase in the relative expression of the mucin 1 (*muc1)* gene in the jejunum of the rats treated with PS-NPs (*p* = 0.001) (Figure 4A) compared with that in the control group. Both of these genes encode proteins that form the mucin layer in the jejunum.

Compared with the control, PS-NPs significantly decreased the expression of genes encoding S100 calcium binding protein B (*s100b)* (*p* < 0.01) and chondroitin sulfate proteoglycan 4 (*cspg4)* (*p* < 0.001) (Figure 3G,H), which are markers of astrocytes and oligodendrocytes, respectively, that constitute the enteric nervous system. The expression of microtubule-associated protein 2 (*map2)* (*p* = 0.181), a marker of enteric neurons, was not significantly affected (Figure 3F).

We also investigated whether prolonged exposure to PS-NPs affects the expression of the genes responsible for the proper function of the intestinal barrier. Analysis of the main barrier markers revealed an increase in the expression of occludin (*ocln*) (*p* < 0.01) and claudin 1 (*cldn1*) (*p* < 0.05) in the rats treated with the PS-NPs (Figure 4B,D). However, no significant effect was noted for the claudin 2 (*cldn2*) (*p* = 0.238) or cadherin (*cdh1*) (*p* = 0.861) gene expression (Figure 4C,E). Moreover, no significant difference in the expression of caspase 3 (*casp3*), which is a marker of the apoptotic process, was detected between the PS-NPs group and the control group (*p* = 0.634) (Figure 4F). The exposure conditions may have been insufficient to induce apoptotic cell death in the jejunal cells of immature rats.

### 2.4. Oxidative Stress and Inflammation Are the Main Pathological Processes Induced by PS-NPs in the Jejunum of Immature Rats

Since the results of the metabolomics analysis revealed changes in the metabolite profiles involved in inflammatory processes, cellular energy maintenance, and oxidative stress between the groups, we explored this issue in detail.

**Figure 5:**
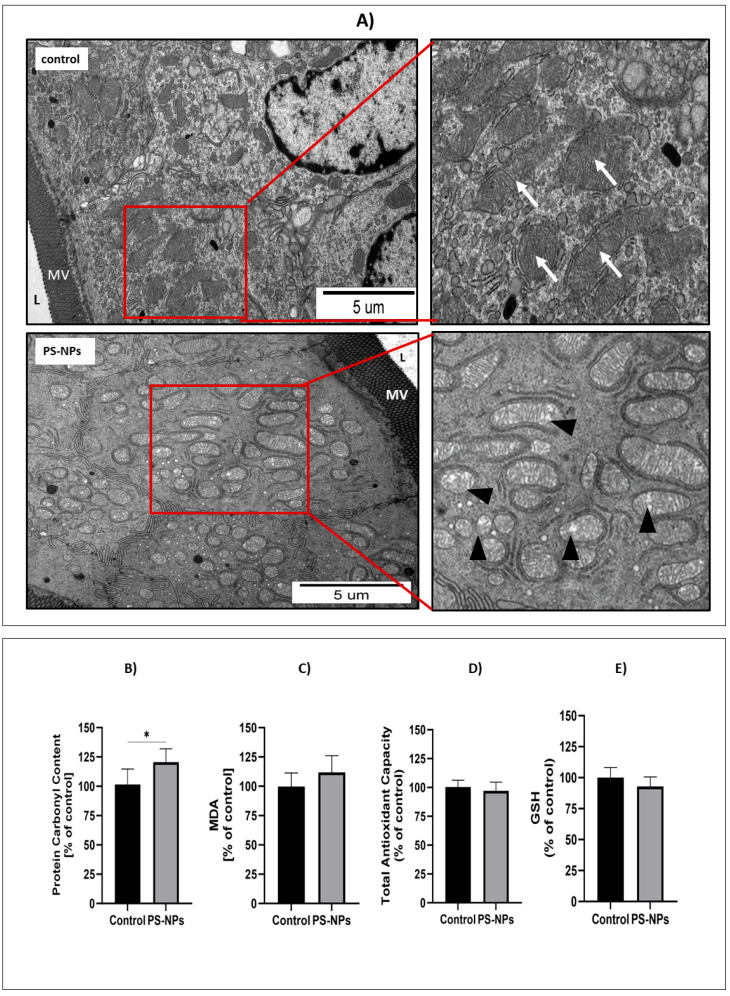
Representative TEM micrographs (**A**) showing morphological features of mitochondria located in enterocytes in the small intestine of control and PS-NPs-exposed immature rats. Insets show normal (arrows) and swollen (arrowheads) mitochondria in specimens obtained from control and exposed animals, respectively; L—intestinal lumen; MV—epithelial “brush border” of microvilli. The graphs show the protein carbonyl group content (**B**), MDA level (**C**), total antioxidant capacity (TAC) (**D**), and GSH level (**E**) in the jejunum of control and PS-NPs-exposed immature rats. The data are presented as the means ± SDs from 6 to 10 independent experiments using distinct animal samples; * *p* < 0.05 (unpaired *t* test).

Morphological, molecular, and biochemical analyses were performed to assess the levels of oxidative stress markers in the jejunum of immature rats exposed to PS-NPs. At the ultrastructural level, we observed that the accumulation of PS-NPs in the intestine induced changes indicative of oxidative stress in mitochondria located in the enterocytic cells. In the samples from the exposed rats, numerous swollen mitochondria were observed, whereas in the control tissue, the mitochondria appeared normal and had a properly organized structure (Figure 5A).

The level of protein carbonyl groups was measured in tissues collected from animals as a marker of irreversible protein oxidation. A statistically significant increase in protein carbonylation was observed in tissues from PS-NPs-exposed animals compared with those from control animals (*p* < 0.05) (Figure 5B). However, the MDA level, which reflects ongoing damage to cellular membranes due to peroxidation, did not change under conditions of PS-NPs exposure (Figure 5C), indicating that PS-NPs-induced oxidative changes involve mainly cellular proteins.

**Figure 6:**
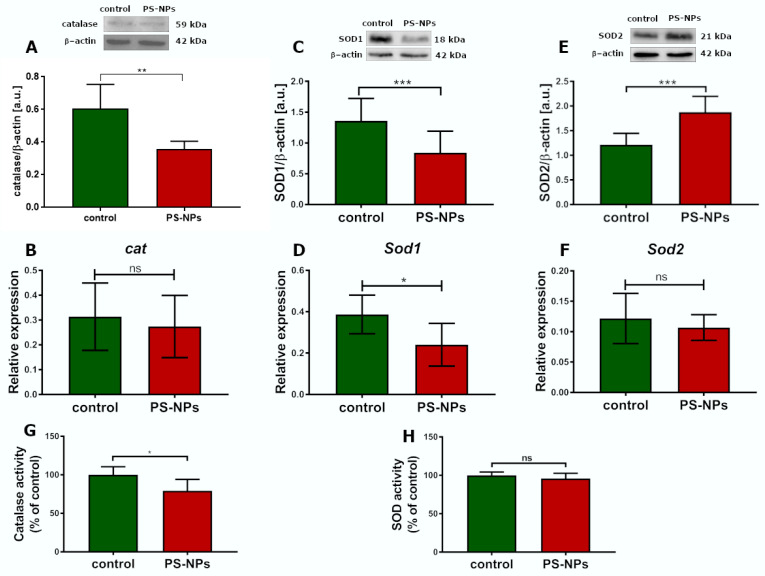
Relative expression of selected antioxidative enzymes in the jejunum of control and PS-NPs-exposed immature rats. Representative immunoblots and graphs showing the expression of the selected oxidative stress response genes at the mRNA and protein levels: CAT (**A**,**B**), SOD1 (**C**,**D**), SOD2 (**E**,**F**), and the activities of CAT (**G**) and total SOD (**H**). The data are presented as the means ± SDs from 6 to 10 distinct animal samples; * *p* < 0.05, ** *p* < 0.01, *** *p* < 0.001; ns—nonsignificant (Student’s *t* test for (**A**–**F**) and unpaired *t* test for (**G**,**H**)).

Oxidative stress occurs in cells when excess free radicals are not properly balanced by antioxidant systems. Therefore, the next step was to assess the total antioxidant capacity (TAC) in the jejunal samples, which was unchanged under conditions of PS-NPs exposure (Figure 5D). Similarly, the level of the main nonenzymatic antioxidant, glutathione (GSH), was not affected by exposure to PS-NPs (Figure 5E). Next, we examined the status of selected enzymatic antioxidant defense systems, such as catalase and superoxide dismutases (SOD1 and SOD2). The relative expression of catalase significantly changed only at the protein level (*p* < 0.01 vs. control) but not at the transcriptional level (Figure 6A,B). Furthermore, the expression of the cytosolic *sod1* gene in the intestine of exposed rats was significantly lower than that in the control group (*p* < 0.05), and a similar profile of changes was observed at the SOD1 protein level (Figure 6C,D). Conversely, the protein level of the mitochondrial SOD2 isoform increased significantly compared with that of the control, whereas the expression of the *sod2* at the mRNA level did not change (Figure 6E,F). The downregulation of CAT and SOD1 proteins indicates possible impairment of the cellular reaction of converting free radicals into H_2_O_2_. Since peroxidases and catalase provide the first line of defense against free radicals, we further checked the activity of these enzymes. We found that catalase activity decreased after exposure to PS-NPs, whereas total SOD activity was unaffected under experimental conditions (Figure 6G,H).

**Figure 7:**
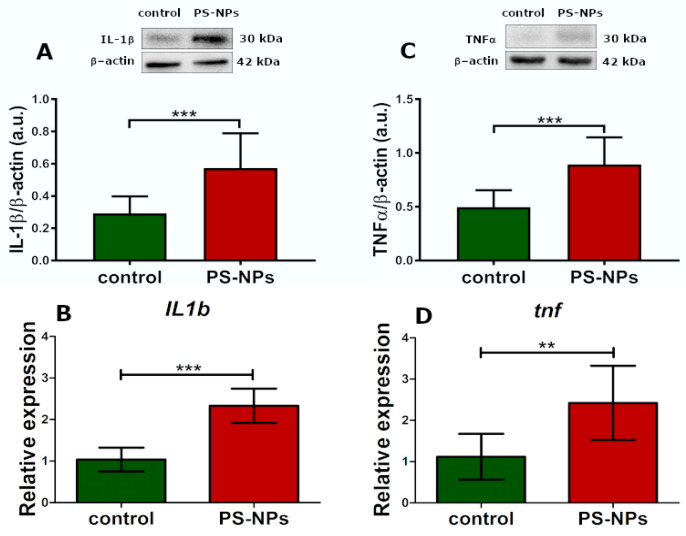
Relative expression of selected proinflammatory cytokines in the jejunum of control and PS-NPs-exposed immature rats. Representative immunoblots and graphs showing the expression of the selected genes encoding proinflammatory cytokines at the mRNA and protein levels: IL-1β (**A**,**B**) and TNFα (**C**,**D**). The data are presented as the means ± SDs from 6 to 10 distinct animal samples; ** *p* < 0.01, *** *p* < 0.001 (Student’s *t* test).

In addition to the presence of markers indicative of oxidative processes, as well as the downregulation and dysfunction of certain enzymatic antioxidant systems that interrogate oxidative stress, we also observed markers of inflammation. The relative protein expression of inflammatory cytokines such as interleukin 1 beta (IL-1β) (*p* < 0.001) and tumor necrosis factor (TNF-α) (*p* < 0.001) was significantly greater in the jejunal samples of the rats exposed to PS-NPs than in those of the control rats (Figure 7A,C). This profile of changes was also confirmed at the transcriptional level. We observed a significant increase in the expression of both these cytokines at the mRNA level (*p* < 0.001 and *p* < 0.01, respectively) (Figure 7B,D).

The results of the molecular and biochemical analyses were largely consistent with the metabolomics results. The relationships of the change profiles in the context of their significance for cellular mechanisms are summarized in Table 3.

## 3. Discussion

### 3.1. Accumulation of PS-NPs in the Jejunum of Exposed Immature Rats and Their Effects on Metabolomics and Gene Expression

Recent evidence suggests that signals from the gut can influence many aspects of health and disease [16]. The intestine is known to be the primary site of nutrient digestion and absorption, as well as the site of microbiota regulation and immune function [17]. It is also the major portal for harmful environmental factors, including microbes and chemicals. Hence, we are interested in whether plastic nanoparticles consumed chronically by immature organisms can impact gut health.

We first examined whether PS-NPs (25 nm) can penetrate the gut cells of immature rats under our experimental conditions. Previous studies have shown that these specific types of nanoparticles are internalized into primary astrocytes via phagocytosis [15]. The current results of the confocal microscopy analysis revealed that PS-NPs accumulate in the crypts and villi and penetrate the intestinal epithelium, where they enter deeper layers of tissue (Figure 1).

We then performed metabolomic analysis via NMR spectroscopy to determine whether there were any differences in the profiles of the hydrophilic and hydrophobic compounds in the jejunal extracts between the control and exposed groups. Metabolomics is a high-throughput detection method that reflects the pathological state through the overall endogenous biochemical phenotype. By analyzing comprehensive metabolic profiles, it is possible to identify metabolites associated with the pathological state and reveal the metabolic pathways in which they are involved [18]. Using two statistical parameters, VIP > 1 or *p* < 0.05, we identified 20 compounds/functional groups (Table 1 and Table 2) that play key roles in pathways responsible for intestinal cell conditions, intestinal barrier integrity, energy status of the cells, oxidative stress, and inflammation (Table 3).

Among the hydrophobic compounds differentiating the experimental and control groups, we detected triacylglycerols (TG) and oleic acid, the concentrations of which were lower in the exposed group, and free cholesterol, lauric/palmitic, and palmitoleic/dodecanoic/arachidonic acids, the concentrations of which were higher in the exposed group than in the control group (Table 2). Exogenous lipids are essential elements of the diet required for lipid homeostasis in the intestine, but also in the whole organism. Hydrolyzed dietary fats are absorbed by enterocytes in the small intestine, where TG, cholesteryl esters, and phospholipids are resynthesized and packaged into lipoproteins called chylomicrons before they enter the bloodstream via the lymph [19]. The changes in metabolite levels observed after PS-NPs exposure suggest disturbed homeostasis of various lipids due to the dysfunction of enterocytes, the primary cells responsible for nutrient absorption. The concomitant downregulation of the *fabp2* gene in the PS-NPs-exposed group is consistent with this concept (Figure 3A), confirming the possible dysfunction of enterocytes. Since FABP2 is a key protein involved in the intracellular transport of long-chain fatty acids in enterocytes, decreased transcriptional levels may indicate impaired absorption by these cells. Consistently, metabolomic analysis revealed changes in the profiles of saturated fatty acids (SFAs), polyunsaturated fatty acids (PUFAs), and monounsaturated fatty acids (MUFAs) in jejunal extracts from PS-NPs-exposed rats compared with those from control rats (Table 3). Furthermore, the decreased expression of *chga*, a marker of enteroendocrine cells that secrete a variety of hormones and peptides involved in the regulation of overall intestinal homeostasis [20], may suggest dysfunction of these cells.

Studies in mice [21] have shown that mucosa exists as a permeable layer in the small intestine that is not attached to the underlying epithelium, is rich in bacteria, and simultaneously contains antibacterial peptides/proteins that limit bacterial contact with the epithelium. Threonine (Thr) is an amino acid that is a component of mucins that coat epithelial surfaces. Our results revealed increased levels of Thr (Table 3) in the jejunum extracts of exposed animals and parallel upregulation of *muc1* gene expression (Figure 4A), which may reflect the compensatory reaction induced to maintain damage to the jejunal mucus layer under exposure to nanoplastics. While increased MUC1 expression may initially be protective, it may also have potential implications. An excessively thick mucus layer may impede efficient nutrient absorption, and changes in MUC1 expression may also affect the interaction between the gut microbiota and the host [22].

We also found decreased levels of myo-inositol, the predominant isomeric form of inositol, which is involved in many cellular functions, particularly as a precursor of phosphatidylinositol (PI) and phosphoinositide (PIP). Thus, depletion of the intracellular content of this metabolite may have a negative effect on the synthesis and cellular availability of PI and PIP [23], which serve as structural elements of biological membranes and are involved in a broad range of biological processes, including cell signaling and membrane dynamics and remodeling. The dysregulation of these processes can lead to detrimental effects [24].

The mammalian intestinal epithelium undergoes rapid and continuous renewal throughout the life of the organism. The stem and progenitor cells that drive this process give rise to all of the differentiated cell types. Notably, the expression of the stem cell gene *lgr5* was significantly upregulated under conditions of PS-NPs exposure compared with that of the control (Figure 3D). This may be the direct response to the presence of PS-NPs or the result of PS-NPs-induced changes in the gut microbiome [8] since microbial metabolites have been reported to be important mediators that regulate intestinal stem cells [25]. Increased expression of *lgr5* may also indicate increased proliferation or activation of stem cells, potentially as a compensatory mechanism induced to repair or replace damaged cells in the intestinal epithelium (downregulation of the *fabp2* and *chga* genes expression). Alternatively, this may indicate a disruption of the stem cell niche, leading to aberrant stem cell activation. This process may be accompanied by alterations in Paneth cell function, as reflected by the upregulation of *lyz1*, which encodes lysozyme 1 (Figure 3E). Secretory Paneth cells, alongside goblet cells, enterocytes, and enteroendocrine cells, are found in the epithelium of the small intestine. Located adjacent to intestinal stem cells, in the crypts of Lieberkühn, these cells are critical to the homeostasis of the immature intestine.

Interestingly, PS-NPs-induced downregulation of genes expressed by glial cells belonging to the enteric nervous system (ENS), such as *s100b* and *cspg4*, was also observed. Enteric glial cells are involved in various cellular processes within the intestine that support neuronal survival and function, maintaining the gut–vascular barrier and exerting immunomodulatory effects [26]. The reduced expression of these genes may indicate that PS-PNs potentially impact the enteric nervous system in immature rats, thereby limiting their supportive functions and the ability of the ENS to adapt to pathological insults.

Our research also revealed significant alterations in several metabolites, indicative of the intestinal barrier status following exposure to PS-NPs, specifically betaine, glycine, alanine, aspartic acid, lysine, and valine (Table 3). A notable finding was the reduced level of betaine, a derivative of glycine metabolism that may compromise small intestine barrier function by decreasing the expression of junctional adhesion molecule-B, occludin, and zonula occludens-1 at the mRNA level [27]. Moreover, recent studies highlight the role of specific amino acids (AAs), including glutamate, alanine, glycine, lysine, serine, and valine, in the production of short-chain fatty acids (SCFAs) through multiple pathways [1]. SCFAs, particularly butyrate, are crucial for maintaining the integrity of the intestinal barrier, as they constitute the main source of energy for enterocytes and have anti-inflammatory properties. Abnormal levels of precursor amino acids, as observed in our study, can lead to unbalanced SCFA production, including alterations in the gut microbiome, impaired nutrient absorption, and gut barrier dysfunction. The metabolic changes observed in many AAs in the present study therefore suggest potential disturbances in overall intestinal function, including barrier integrity. To further investigate intestinal permeability, we analyzed the expression of genes encoding key components of tight junctions, such as *ocln*, *cldn1*, *cldn2*, and *cdh1* (occludin, claudin-1, claudin-2, and cadherin-1/E-cadherin). Occludin and claudin-1 are particularly vital components of tight junctions, forming the basic seal between intestinal epithelial cells and controlling paracellular permeability [28]. Interestingly, our qRT-PCR analysis revealed that expression of both genes was upregulated. The observed overexpression may be an early adaptive mechanism in response to initial barrier damage in the presence of PS-NPs, where tight junctions become compromised, and increased gene expression is an attempt to counteract this damage. Alternatively, PS-NPs may also directly influence the transcriptional machinery of jejunal cells, leading to altered gene expression. The correlation between altered metabolites and changes in gene expression indicates multifaceted disruption of the intestinal barrier. While an altered amino acid profile, particularly betaine, may indicate an environment less supportive of barrier integrity, the concomitant upregulation of genes encoding tight junction proteins suggests an active response of the cellular machinery to maintain the barrier. Reduced betaine/choline directly affects the availability of methyl donors and essential building blocks for tight junction proteins, potentially leading to reduced function even if their expression is initially increased as a compensatory measure. Importantly, the effect of PS-NPs on the intestine is likely not a simple, single mechanism. The observed results may reflect the complex interplay of multiple factors, including inflammation, oxidative stress, and changes in the gut microbiome.

These factors can influence gene expression and the functional integrity of junctional complexes, even if their genes are upregulated. Further studies are needed to examine functional integrity (e.g., measuring transepithelial electrical resistance) to determine whether increased expression actually translates into the most robust barrier or is an ineffective attempt at repair.

### 3.2. PS-NPs Induced Oxidative Stress and Inflammation in the Jejunum of Immature Rats

The intestine is the active site of the metabolic reactions of endogenous amino acids. In addition, essential exogenous amino acids that must be obtained from food undergo metabolic reactions, producing molecules important for overall metabolic function [29]. Compared with those of the control samples, the levels of the following amino acids increased significantly after PS-NPs exposure: methionine (Met), lysine (Lys), alanine (Ala), threonine (Thr), valine (Val), and glycine (Gly), as did the levels of guanidinoacetic (GAA) and ketoglutaric/ketoisovaleric acids. These AAs are involved in a variety of physiological metabolic pathways, and their altered homeostasis may disturb cellular energy pathways, oxidative stress and inflammation. For example, Met, an essential amino acid, is an important precursor of cysteine (Cys), the key substrate for the synthesis of the antioxidant glutathione. In turn, a metabolite of Met, S-adenosylmethionine, regulates the expression of amino acid transporters located in the intestinal epithelium, which can be affected by inflammatory processes [30]. Guanidinoacetic acid (GAA), synthesized from arginine and glycine, is a key precursor of creatine, thereby supporting energy metabolism by regulating ATP homeostasis in cells [29]. In turn, nicotinic acid (niacin, vitamin B3) deficiency, which we observed in PS-NPs-exposed rats (Table 2 and Table 4), may also lead to inflammation and barrier dysfunction by modulating intestinal cell proliferation and the expression of occludin and claudin-1 at the mRNA level [31].

Disruption of amino acid metabolism is a frequently occurring mechanism underlying the toxicity of various environmental pollutants, including metals and nanoparticles [32]. The altered profile of the abovementioned metabolites is consistent with the results indicating the upregulation of genes encoding proinflammatory cytokines such as IL-1β and TNFα and the downregulation of certain antioxidative enzymes. IL-1β is a potent proinflammatory cytokine that plays a key role in initiating and amplifying the inflammatory response. Therefore, the overexpression of the *Il-1β* gene undoubtedly indicates an ongoing inflammatory process in the jejunum of exposed rats, which is further supported by the increased expression of *tnfα*. This observation is in agreement with a previous report that reviewed the health outcomes of nano/microplastics relevant to colitis and inflammatory bowel disease [33]. The inflammatory response may be induced by various mechanisms, including direct activation of immune cells, such as macrophages and dendritic cells, by PS-NPs or induction of dysbiosis of the gut microbiota, leading to an imbalance that favors proinflammatory bacteria.

The overexpression of proinflammatory cytokines in parallel with the increased level of lysozyme at the mRNA level (*lyz1*) may also explain the increased activity of Paneth cells in response to the pathological influence of PS-NPs. The secretory panel of these cells includes proteins and peptides that modulate the microbiome and mediate the inflammatory response, including LYZ1, TNF-α, IL-17A, IL-1β, angiogenin-4, MMP-7, xanthine oxidase, IgA, and lipokines [34].

As mentioned above, we also observed the upregulation of the *muc1* gene encoding mucin 1 glycoprotein, which forms a protective barrier on the surface of epithelial cells against pathogens, toxins, and other harmful substances and plays a role in cell signaling and immune regulation. The observed increase in *muc1* expression may be a compensatory response to the harmful effects of PS-NPs, which trigger MUC1 production to strengthen the intestinal barrier and protect the underlying tissue. An alternative explanation is that the observed upregulation of *muc1* is due to the oxidative stress induced in jejunal cells by PS-NPs, since this protein has been shown to have a protective effect against oxidative stress by decreasing intracellular ROS levels and attenuating ROS-induced apoptosis [35].

Indeed, we observed increased levels of protein carbonyl groups in the jejunum of exposed rats (Figure 5B), a parameter indicative of irreversible oxidative damage to cellular proteins. Moreover, decreased expression and/or activity of certain enzymatic antioxidant defense systems (SOD1 and CAT) (Figure 6), which are crucial for restoring redox homeostasis, may contribute to oxidative stress [36]. These findings are consistent with the results of Han et al. [37], who reported that exposure to nanoplastics increased protein carbonylation and concomitantly reduced intestinal antioxidant defense mechanisms in aquatic crustaceans. The role of oxidative stress in the mechanism of jejunal damage and gut barrier disintegration caused by various environmental factors has been previously stressed [38].

Similarly, the edematous changes revealed in the mitochondria of PS-NPs-exposed animals at the ultrastructural level (Figure 5A) undoubtedly indicate potential damage leading to energy failure and oxidative stress. Mitochondria are structures whose efficient function is highly important for cells. They play a variety of roles, including guarding reactive oxygen production and redox homeostasis, and mitochondrial dysfunction has a primary role in the pathogenesis of many diseases [39]. In parallel with the morphological alterations, we observed elevated levels of alpha-ketoglutarate (AKG), an intermediate in the Krebs cycle that links amino acid metabolism to glucose oxidation, which may suggest reduced utilization of this molecule by dysfunctional mitochondria. AKG oxidation also supplies large amounts of ATP and modulates the redox state of cells in the small intestine, improving intestinal mucosal integrity and nutrient absorption [40]. Therefore, the limited potential of its use by the cell leads to energy demand.

## 4. Materials and Methods

### 4.1. PS-NPs and Experimental Design

Polystyrene nanoparticles (PS-NPs; 25 nm) labeled with a fluorochrome with a red fluorescence spectrum and an excitation/emission wavelength of 545/566 nm were purchased from Lab261 (Palo Alto, CA, USA). The batches of PS-NPs were additionally characterized in detail as previously described [14,15] and the measurements of zeta potential showed that under physiological conditions (37 °C and pH 7.4) PS-NPs remain stable in solution and do not agglomerate.

All experimental procedures involving animals were carried out in accordance with the EU Directive for the Care and Use of Laboratory Animals (Directive 2010/63/EU) and were approved by the II Local Experimental Animal Care and Use Committee in Warsaw (WAW2/043/2022).

Animals were purchased from the Animal House of the Mossakowski Medical Research Institute Polish Academy of Sciences, Warsaw, Poland (MMRI, PAS). On postnatal day 14 (PND 14), pups from 3 pregnant dams (total number *n* = 28) were randomly assigned to the experimental and control groups. The experimental group was administered 1.0 mg/kg b.w. of PS-NPs via gastric tube for 21 consecutive days. The appropriate volume of PS-NPs solution was adjusted daily depending on changes in body weight, with a maximum volume not exceeding 200 µL. The control group received a corresponding volume of 0.9% NaCl. The dose of PS-NPs was calculated on the basis of available literature data, indicating that the estimated daily intake of plastic particles for humans ranges from 2.4 to 700 mg, which corresponds to a dose of 0.04–11.7 mg/kg b.w. for humans weighing approximately 60 kg [12]. During the first week of exposure, the pups were returned to their cages after receiving the solutions. They stayed with their mothers until they were able to eat on their own, after which they were separated and placed in individual cages. The animals were sacrificed via decapitation at postnatal day 35 (PND 35) to obtain intestinal samples or were anesthetized and perfused for microscopic analysis. Since the jejunum is the part of the small intestine that absorbs most of the nutrients from food, such as vitamins, minerals, carbohydrates, fats, and proteins, we used this part of the tissue for further assays.

### 4.2. Sample Preparation for NMR Examinations

The collected jejunal samples were gently rinsed with saline to remove the intestinal contents, avoiding damage to the villi, and frozen at −20 °C until extraction for measurement. After thawing, the samples were rinsed again with saline, dried on filter paper and weighed. Samples weighing 42 ± 14 mg were homogenized and placed in polypropylene (PP) tubes in extraction mixtures consisting of chloroform/methanol/36% HCl at a volume ratio of 1:2:0.1 (total volume 1875 µL). The samples were subsequently shaken at room temperature (RT) at 1400 rpm for 30 min using a Biosan Multispeed vortex msv 3500. After this, 625 µL of chloroform was added, and the samples were shaken again as described above. This procedure was repeated after 625 µL of H_2_O_2_ was added. After the extraction step, the samples were centrifuged at the following parameters: 4 °C and 2170× *g* for 30 min to obtain three layers: the upper methanol–water layer containing hydrophilic compounds, the lower chloroform layer containing hydrophobic compounds and the middle layer containing proteins. The lower and upper layers were transferred separately to new PP tubes and evaporated under nitrogen. The dry masses were dissolved with 600 µL of deuterated water (D_2_O) and 700 µL of deuterated chloroform (CDCl_3_) for hydrophilic and hydrophobic compounds, respectively, and then transferred to quartz tubes for NMR spectrometric measurements.

### 4.3. NMR Spectroscopy Measurements

The pH of each sample was adjusted to 7.5 ± 0.2 with HCl. Three-trimethylsilyl propionic acid (TSP) (1 mM) was used as an internal standard to normalize all the spectra. All NMR spectra of the hydrophilic compounds were obtained at 25 °C using an Inova 400 MHz spectrometer (Varian Inc., Palo Alto, California, USA). For the measurements of the spectra of the hydrophilic compounds, a single-pulse sequence with water presaturation, 128 transients and a repetition time of 10 s was used. A 0.5 line broadening, baseline and phase correction were applied to each spectrum via software implemented in the spectrometer. The hydrophobic compounds were measured via a single-pulse sequence at 20 °C with 128 transients and a repetition time of 5 s.

All the spectra were both baseline- and phase-corrected prior to analysis. Signal assignments were performed via the database of reference compound spectra and literature data, taking into account the correction for the modified extraction method [41]. For further statistical analyses, we selected 45 hydrophilic and 26 hydrophobic most isolated NMR signals, which represent all assigned and unassigned compounds, and their magnitudes were measured and normalized to those of TSP or CHCl3 prior to statistical analyses. All the measurement results were normalized to the wet weight of each sample.

### 4.4. Visualization of PS-NPs in the Jejunum by Confocal Microscopy

The frozen jejunum samples (prepared as described above) were cut into 20 μm sections on a cryostat (Leica CM1860 UV, Leica Biosystems, Nussloch, Germany), placed on slides (Thermo Fisher Scientific, Waltham, MA, USA), rinsed in PBS and incubated in 4% paraformaldehyde for 20 min. Next, the samples were washed with PBS and incubated in 10% goat serum in PBS containing 0.25% Triton X-100 and 0.1% bovine serum albumin for 60 min at RT. Next, the cell nuclei were stained with Hoechst 33258 (Sigma–Aldrich, St. Louis, MO, USA; 1 µg/mL). A positive control was established by the incubation of sections obtained from control animals with 0.5 µg of labeled PS-NPs for 5 min. Three animals per group and images from six sections per animal were taken for analysis. The fluorescence intensity was measured in similar regions of interest (ROIs). To visualize the presence of PS-NPs in the tissue, images were taken using an LSM780/Elyra PS.1 confocal laser scanning microscope with ZEN 2.6 software (Carl Zeiss, Jena, Germany) at the Laboratory of Advanced Microscopy Techniques, MRI PAS.

### 4.5. Transmission Electron Microscopy (TEM) Analysis

The animals were deeply anesthetized with a ketamine and xylazine mixture and perfused first with 0.9% NaCl in 0.01 M sodium–potassium phosphate buffer (pH 7.4) and then with a fixative solution (2% paraformaldehyde and 2.5% glutaraldehyde in 0.1 M cacodylate buffer; pH 7.4). Jejunal samples were collected from the control and experimental animals, placed in the abovementioned ice-cold fixative and then subjected to a routine method of tissue processing for TEM. Briefly, the tissues were postfixed in 1% OsO4 solution and dehydrated in an ethanol gradient. After being embedded in epoxy resin (Epon 812), ultrathin sections were cut and stained with 9% uranyl acetate and lead nitrate for examination via transmission electron microscopy (TEM) (JEM-1200EX, Jeol, Tokyo, Japan) with the digital camera MORADA and iTEM 1233 software (Olympus Soft Imaging Solutions, GmbH, Münster, Germany).

### 4.6. Analysis of Relative Gene Expression by qRT–PCR

#### 4.6.1. RNA Isolation

Total RNA from jejunum tissue was isolated using Bead-beat Total RNA Mini Kit (A & A Biotechnology, Gdynia, Poland) or TRI reagent (Sigma–Aldrich, St. Louis, MO, USA). The RNA concentration was determined using a DS-11 FX spectrophotometer (Denovix, Wilmington, Denmark).

#### 4.6.2. RT

The RNA was then transcribed into cDNA by reverse transcription–polymerase chain reaction (RT) using the high-capacity RNA–cDNA™ Kit (Applied Biosystems, Forest City, CA, USA) following the manufacturer’s instructions.

#### 4.6.3. SYBR GREEN Gene Expression Assays

For qRT–PCR analysis, 10 ng of cDNA was mixed with 0.25 μM forward and reverse primers, along with 12.5 μL of iTaq™ Universal SYBR^®^ Green Supermix (Bio-Rad, Hercules, CA, USA). The samples were loaded into Roche 96-well qRT–PCR plates with optical foils (BLIRT S.A., Gdansk, Poland) and subjected to the following thermal cycling conditions: an initial denaturation step at 95 °C for 3 min, followed by 45 cycles of denaturation at 95 °C for 10 sec and annealing/extension at 58 °C for 1 min, performed on a LightCycler 96 (Roche, Basel, Switzerland). Relative gene expression (*fabp2*, *chga*, *lgr5*, *lyz1*, *muc2*, *muc1*, *s100β*, *cspg4*, *map2*, *Il1b*, *tnf*, *Il23a*, *ocln*, *cldn1*, *cldn2*, *cdh1*, *casp3*) was calculated by the ΔΔCt method [42], and the reference gene (*sdha*) was selected from the reference gene panel (*sdha, rplp0, hprt1, ubc, b2m, tbp*) using the comprehensive online tool RefFinder web-based, as described by Augustyniak et al., 2019 [43]. The sequences of all the primers used for the qRT–PCR experiments are presented in Table 4.

**Table 4:** Primers and TaqMan probes used for qRT–PCR analysis.

Gene Symbol	Gene Name	GenBank Number	Primer Sequence	Amplicon Length
*fabp2*	*Fatty acid binding protein 2*	NM_013068.1	ATCATGGCATTTGATGGCACTTG	150
			TCCTGTGTGATCGTCAGTTTCAA	
*chga*	*Chromogranin A*	NM_021655.2	CGGAAGTATTTGAGAACCAGAGC	113
			ATCCTGTTGCCCCTTGTCAG	
*lgr5*	*Leucine-rich repeat-containing G protein-coupled receptor 5*	NM_001106784.1	ATACTGTCACTGTGAGCTGGATG	132
			TGACTGATGTTGTTCATACTGAGGT	
*lyz1*	*Lysozyme*	NM_012771.3	AAGGAATGGGATGTCTGGCT	127
			CCCATAGTCGGTGCTTTGGT	
*muc2*	*Mucin 2*	NM_022174.1	GATGTGTGGGACCGGACAAT	118
			GCACACTTCTTTGGTTGGCA	
*muc1*	*Mucin 1*	NM_001398538.1	CTCGGAAGTCAATGTGAATGAGATG	
			CAATGAGATAGACGATGACCAAAGC	131
*s100β*	*S100 calcium-binding protein B*	NM_013191.2	TGTCTACCCTCCTAGTCCTCG	94
			CCTTCTCCAGCTCAGACATCC	
*cspg4*	*Chondroitin sulfate proteoglycan 4*	NM_031022.2	CTCCAGTTCTCCACATCGCA	119
			TTTTGTCCCAGGGCAAGTCT	
*map2*	*Microtubule-associated protein 2*	NM_013066.1	GCCGGGGCCATGATCTTTC	80
			GTAATCATCTCCTTCATCCATCGTT	
*Il1b*	*Interleukin 1 beta*	NM_031512.2	GCTATGGCAACTGTCCCTGA	134
			TCTGGACAGCCCAAGTCAAG	
*tnf*	*Tumor necrosis factor*	NM_012675.3	ATGGGCTCCCTCTCATCAGT	106
			GCTTGGTGGTTTGCTACGAC	
*Il23a*	*Interleukin 23 subunit alpha*	NM_130410.2	AAAAGTGACGTGCCCCGTAT	98
			AGACCTTGGCGGATCCTTTG	
*ocln*	*Occludin*	NM_031329.3	GCAGTGAACAAGCTGTGTCTAAA	179
			CGGCTAAAACAGACCAAACTGG	
*cldn1*	*Claudin 1*	NM_031699.3	GACTGCTCAGGCCATCTACG	132
			ACCATCAAGGCTCTGGTTGC	
*cldn2*	*Claudin 2*	NM_001106846.2	CGAGAAAGAACAGCTCCGTTT	105
			TCACAGTGTCTCTGGCAAGC	
*cdh1*	Cadherin 1	NM_031334.1	ATTACAAGTTCCCGCCATCCTT	148
			ATACACATTGTCCCGGGTATCG	
*casp3*	*Caspase 3*	NM_012922.2	GGAGCTTGGAACGCGAAGAAAAG	135
			CTGCTGTCCAGATATATTCCAGAGT	
*sdha*	*Succinate dehydrogenase complex flavoprotein subunit A*	NM_130428.1	GTATTTCGCACTGGATCTTCTGATG	97
			CTTTGCTCTTATTCGGTGTATGGAC	
*actb*	*Actin, beta*	NM_031144.3	TACAACCTTCTTGCAGCTCCTC	200
			TGACCCATACCCACCATCACAC	
*rplp0*	*Ribosomal protein lateral stalk subunit P0*	NM_022402.2	CATCAATGGATACAAAAGGGTCCTG	271
			TCTTTCTCAAATTAAGCAGGCTGAC	
*hprt1*	*Hypoxanthine phosphoribosyltransferase 1*	NM_012583.2	CTTCCTCCTCAGACCGCTTTTC	169
			ATCAGTCCATGAGGAATAAACACCT	
*ubc*	*Ubiquitin C*	NM_001399781.1	AAGATACTCGTACCTTTCTCACCAC	88
			AAAACTAAGACACCTCCCCATCAAA	
*b2m*	*beta-2 microglobulin*	NM_012512.2	ATGTTAGGATGAAAGCCCAGGTATT	141
			CCAACAGAATTTACCAGGAAACACA	
*tbp*	*TATA box binding protein*	NM_001004198.1	CACCGTACATCTCAGCTGCTTC	134
			ATCTCCTTAGAAACGTCTTCGACTT	
**TaqMan probes**				
**Gene symbol**	**Gene name**	**Chromosome Location**	**Assay ID**	**Manufacturer**
*sod1*	*Superoxide dismutase 1*	Chr.11: 30363282–30368858 on Build Rnor_6.0	Rn00566938_m1	Thermo Fisher Scientific (Warsaw, Poland)
*sod2*	*Superoxide dismutase 2*	Chr.1: 47914757–47921587 on Build Rnor_6.0	Rn00690588_g1	Thermo Fisher Scientific (Warsaw, Poland)
*cat*	*Catalase*	Chr.3: 93379872–93412058 on Build Rnor_6.0	Rn01512560_m1	Thermo Fisher Scientific (Warsaw, Poland)
*gpx1*	*Glutathione peroxidase 1*	Chr.8: 117117430–117118528 on Build Rnor_6.0	Rn00577994_g1	Thermo Fisher Scientific (Warsaw, Poland)
*gpx4*	*Glutathione peroxidase 4*	Chr.7: 12516357–12519154 on Build Rnor_6.0	Rn00820818_g1	Thermo Fisher Scientific (Warsaw, Poland)

#### 4.6.4. TaqMan Gene Expression Assays

Quantitative PCR (qRT–PCR) was performed on a Light Cycler^®^ 96 System (Roche Diagnostics GmbH, Mannheim, Germany) using 5 µL of the RT product in a total reaction volume of 20 µL with the TaqMan Gene Expression Assay Kit (Thermo Fisher Scientific, Warsaw, Poland). The amplification protocol consisted of an initial denaturation step at 95 °C for 10 min, followed by 40 cycles of 15 s at 95 °C and 60 s at 60 °C. Each sample was analyzed in triplicate. Gene expression levels (*cat*, *sod1*, and *sod2*) were quantified using the ΔΔCt method, with actin beta (*actb*) as the reference gene. The information about the TaqMan probes purchased from Thermo Fisher Scientific is presented in Table 4.

### 4.7. Western Blot Analysis

Protein analyses by Western blot were performed according to standard procedures. The detailed data are described in the Supplementary Materials. The original blots are also included and marked as Figures S1–S5.

### 4.8. Measurements of Oxidative Stress Markers

Oxidative stress markers were tested using appropriate kits according to the procedures recommended by the manufacturers: an MDA assay kit Merck (Warsaw, Poland), a protein carbonyl content assay (Merck), a total antioxidant capacity (TAC) assay (Cayman Chemical, Warsaw, Poland), a glutathione (GSH) assay kit (Merck, Warsaw, Poland), a catalase colorimetric activity kit (Thermo Fisher Scientific, Warsaw, Poland) and a superoxide dismutase (SOD) activity assay kit (Merck, Warsaw, Poland). More information about the procedures is provided in the Supplementary Materials. 

### 4.9. Statistical Analysis

For the NMR results, univariate statistical analyses and one-way ANOVA tests followed by Dunn’s correction were performed via SIGMA Plot 12.5 software (Systat Software, Inc., San Jose, CA, USA). A *p*-value lower than 0.05 was considered significant. The process of multivariate statistical analysis (MVA) was described in detail in our previous publication [44]. Briefly, for the analysis, we used a supervised method, orthogonal partial least squares discriminant analysis (OPLS-DA), in which the X-matrix (independent variables) represents all the data obtained from the NMR spectra and the Y-matrix (dependent variables) represents the experimental groups [45]. Data (X-matrix) are means centered and Pareto scaled. To validate the model, a jackknife test was implemented. The results of the constructed models are outlined in terms of goodness of fit to the data: R^2^ for the overall model and Q^2^ for the predictive quality. The theoretical maximum score is 1, which indicates perfect prediction, and an R2 greater than 0.5 is considered good. For the OPLS component to be considered significant, Q^2^ must be significantly greater than zero, with values equal to or greater than 0.5 generally considered good. The most important compounds for classification were identified using the variable importance for projection (VIP) score. VIP values greater than 1 were considered significant. OPLS-DA was performed via the SIMCA software package, ver. 15, Sartorius Stedim Data Analytics AB, Sweden [46].

For the statistical analysis of the data obtained from the qRT–PCR, WB, and biochemical assays, GraphPad Prism 5.0 and 10.0 software (San Diego, CA, USA) was used. For the qRT–PCR data, the normality of distribution was assessed using the Shapiro–Wilk test. Depending on the results, the unpaired Student’s *t* test (for normal distribution and variance equality), the unpaired Student’s *t* test with Welch’s correction (for normal distribution and lack of variance equality), or the Mann–Whitney test (for lack of normal distribution) was used. For other data, unpaired Student’s *t* tests or Mann–Whitney tests were used, as indicated under the respective figures. The data in the bar graphs are presented as the means ± SDs from three to ten distinct animal samples, as stated in the figure legends. A *p*-value < 0.05 was considered statistically significant.

## 5. Conclusions

In this study, we investigated the jejunal effects of prolonged exposure to PS-NPs in immature rats. Our findings demonstrate that PS-NPs are absorbed in the small intestine and accumulate in the epithelium and subepithelial layer, where they exhibit pro-oxidative and proinflammatory potential, as shown by metabolomics, ultrastructural, molecular, and biochemical analyses. These results support the hypothesis that oxidative stress and inflammation are the main mechanisms underlying the developmental toxicity of PS-NPs, which may disturb gut homeostasis.

Moreover, molecular analysis also revealed that the PS-NPs significantly altered the expression of many other genes involved in key intestinal functions. The observed downregulation of genes associated with nutrient absorption, enteroendocrine function, and the enteric nervous system points to the potential of PS-NPs to affect numerous processes/mechanisms, thereby disrupting the proper functioning of the intestine. The positive aspect is that we concomitantly observed the activation of compensatory mechanisms, such as the upregulation of certain genes encoding intestinal barrier proteins and a regenerative response of intestinal stem cells. These findings indicate the potential of young organisms to adapt partially to the damaging effects of PS-NPs; however, they highlight the importance of further detailed research to elucidate the precise mechanisms by which PS-NPs interact with the intestinal epithelium of immature organisms and the potential long-term consequences of PS-NPs exposure to gut health.

## Figures and Tables

**Table 1:** Changes in the NMR signal intensity of hydrophilic compounds in jejunum extracts obtained from PS-NP-exposed immature rats expressed as percentages of the control, *p* values, and VIP values.

Hydrophilic Metabolite	PS-NPs vs. Controls [%]	*p*	VIP
NAD	61	0.248	
Nicotinic acid	55	**0.017**	
Purine	75	0.303	
IMP/Inosine/Adenosine	137	0.126	
ITP/AMP/IMP/Nicotinic acid	79	0.565	
Hypoxanthine	100	0.662	
Quinolinic acid/Adenine xanthine/Purine	59	0.247	
Uridine	101	0.931	
Guanine	50	0.537	
Phenylalanine	208	0.177	
Cytidine/Cytosine	182	0.126	
Histamine/Serotonin	123	0.387	
Tyrosine	174	0.223	
Fumarate	66	0.322	
AMP/IMP	119	0.547	
Uracil	91	0.429	
α-D glucose	213	0.429	
β-D-glucose	207	0.110	
Guanidinoacetic acid	183	0.229	**2.02**
Myo-inositol	73	0.177	**1.12**
Glycine	156	0.247	**1.20**
Taurine	80	0.380	
Trimethylamine oxide (TMAO)/Betaine	80	0.534	**2.11**
Taurine/Myo-inositol	95	0.829	
Phosphorylcholine	51	0.128	**2.09**
Choline	89	0.329	**2.52**
Ethanolamine/Arginine/Cysteine/Ornithine	95	0.247	
Creatinine	71	0.340	
Creatine	69	0.292	**1.24**
α-ketoisovaleric acid/ α-ketoglutaric acid	190	0.126	**1.42**
Aspartic acid	49	**0.013**	
Glutamine GLN	177	0.217	
Glutamic acid GLU	142	0.429	
Proline	159	0.082	
α-ketoglutaric acid	124	0.530	
Methionine	145	0.389	**1.17**
Arginine	164	0.265	
Lysine	175	0.126	**1.20**
Alanine	180	0.126	**2.39**
Lactate	100	0.989	
Threonine	147	0.429	**1.07**
3-hydroxyisovalerate/ Methylmalonate/Lipid	76	0.429	
α-ketoisovaleric aid	166	0.329	
Valine	157	0.302	**1.35**
Isoleucine/Leucine	192	0.247	

Variable significance in the projection values (VIPs) and *p* values of identified metabolites between the control and PS-NP-exposed groups at the discovery stage (VIP > 1, *p* value < 0.05). The results that are statistically significant are presented in bold.

**Table 2:** Changes in the NMR signal intensity of hydrophobic compounds in jejunum extracts obtained from PS-NP-exposed immature rats expressed as percentages of the control, *p* values, and VIP values.

Hydrophobic Metabolite/Functional Group	PS-NPs vs. Controls [%]	*p*	VIP
16-hydroxyestradiol	145	0.324	
Estriol	107	0.945	
Testosterone	201	0.269	
Phosphatidylcholine (PC)/Phosphatidylethanolamine (PE)/Sphingomyelin (SM)	103	0.928	
1,2-Diacyloglycerol (DAG)/2-Monoacyloglycerol (MAG)	98	0.445	
Phosphatidylcholine/Phosphatidylethanolamine	81	0.445	
Triglycerides (TG)	45	0.132	**1.23**
1,3-DAG	120	0.534	
SM	95	1.0	
PC	112	0.712	
1-MAG or 1,2-DAG	108	0.826	
PC/SM	78	0.534	
PUFA (arachidonic, alfa-linolenic, DHA)	88	0.330	
Linoleic acid	97	0.897	
Saturated fatty acid (FA), polyunsaturated FA (PUFA) and monounsaturated FA (MUFA) (lauric, myristic/dodecanoic/palmitic, arachidonic, alfa-linolenic/oleic)	92	0.769	
Hexanoylglycine	117	0.836	
Oleic acid	76	0.394	**1.65**
Vaccenic acid	102	0.836	
Palmitoleic acid	90	0.295	
Free cholesterol and 1,3-DAG, 1-MAG, and FA (dodecanoic acid)	100	0.628	
Saturated FA and PUFA (lauric/palmitic)	106	0.808	**3.41**
Cholestenol	114	0.534	
Saturated FA, PUFA, and MUFA (dodecanonic, palmitic/arachidonic/palmitoleic, oleic,	107	0.445	**1.72**
Pelargonic acid (nonanoic acid)	99	0.628	
24S- hydroxycholesterol	129	0.366	
Free cholesterol	120	0.376	**1.40**

Variable significance in the projection values (VIPs) and *p* values of identified metabolites between the control and PS-NPs-exposed groups at the discovery stage (VIP > 1, *p* value < 0.05). The results that are statistically significant are presented in bold.

**Table 3:** Relationship between metabolomic and gene expression profiles in the jejunum of immature rats exposed to PS-NPs with respect to cellular mechanisms and metabolic pathways.

Compound/ Functional Group	Direction of Changes vs. Control	Metabolic Pathway	Probable Mechanism/Process	Marker Expression *	Direction of Changes vs. Control
Nicotinic acid	↓	Nicotinate and nicotinamide metabolism	-Intestinal inflammation -Barrier function	IL1β/*Il1β* TNFα/*tnfα* *lgr5* *muc1* *cldn1* *ocln*	↑/↑ ↑/↑ ↑ ↑ ↑ ↑
Guanidinoacetic acid Threonine Glycine Methionine Creatine TMAO/Betaine	↑ ↑ ↑ ↑ ↓ ↓	Glycine, serine, threonine and cysteine, methionine metabolisms	-Intestinal barrier damage -Cellular energy disturbance -Oxidative stress -Effect on mucosal cells -Disturbance of the intestinal protein synthesis		
Myo-inositol	↓	Inositol phosphate metabolism	-Nerve conduction impairment -Axonal degeneration, demyelination	*chga* CAT/*cat* SOD1/s*od1* SOD2/s*od2* *s100b* *cspg4* *fabp2*	↓ ↓/- ↓/↓ ↑/- ↓ ↓ ↓
Phosphoryl choline choline	↓ ↓	Glicero phospholipids metabolism	Oxidative stress		
Ketoisovaleric acid/ ketoglutaric acid	↑	TCA cycle	-Cellular energy disturbance -Oxidative stress -Effect on enterocytes -Absorption disorders		
Aspartic acid Alanine	↓ ↑	Alanine, aspartate and glutamate metabolism	-Intestinal barrier damage -Absorption disorders -Impact on intestinal microbiota	*cldn1* *ocln* *lgr5* *muc1* *lyz1* *fabp2*	↑ ↑ ↑ ↑ ↑ ↓
Lysine Valine	↑ ↑	Valine, leucine, isoleucine biosynthesis/ degradation	-Impact on intestinal microbiota -Intestinal barrier damage -Absorption disorders		
Triglycerides Free cholesterol	↓ ↑	Glicerolipids and cholesterol metabolisms	Effect on enterocytes		
Oleic acid Lauric/palmitic acid Dodecanoic/ palmitic/arachidonic/ oleic acid	↓ ↑ ↑	Fatty acids biosynthesis/ metabolism	Impact on enterohepatic circulation		

* The expression of the marker at the mRNA level is indicated in lower-case italics, and at the protein level, with capital letters; changes: ↑—increased; ↓—decreased; “-”—unchanged.

## Data Availability

The data that support the findings of this study are available from the corresponding author upon reasonable request.

## Supplementary Materials

The following supporting information can be downloaded at: https://www.mdpi.com/article/10.3390/ijms26157207/s1, This section describes the details of the Western blot analysis, including the original scans of the full-length gels and the details of the measurements of oxidative stress markers.
