# Supplementary material for: Nanoplastic Disrupts Intestinal Homeostasis in Immature Rats by Altering the Metabolite Profile and Gene Expression
Source: Int J Mol Sci. 2025 Jul 25;26(15):7207. doi: 10.3390/ijms26157207 (PMC12346606; doi:10.3390/ijms26157207)
Supplement: Supplementary file 1 [file ijms-26-07207-s001.zip › ijms-3728261-supplementary.pdf]

Supplementary material

Western blot analysis

For Western blot analysis, the intestine was homogenized in RIPA lysis buffer (Thermo Fisher Scientific) supplemented with proteinase (Merck, 1:100) and phosphatase (Merck, 1:100) inhibitors. The lysates were centrifuged at 13,000 × *g* for 20 min at 4°C, and the supernatants were collected. Total protein concentrations were assessed using the Pierce BCA protein assay kit (Thermo Fisher Scientific; Western). Equal amounts of protein samples (50 μg) were separated on 15% or 10% sodium dodecyl sulfate–polyacrylamide gel electrophoresis (SDS–PAGE) and then transferred onto nitrocellulose membranes (Amersham™ Protran™ P 0.45 PVDF). After blocking in buffer containing phosphate-buffered saline, Tween-20 and 5% nonfat dried milk, the membranes were incubated at 4°C overnight with the following primary antibodies: rabbit anti-catalase polyclonal antibody (Invitrogen, 1:500), rabbit anti-SOD1 polyclonal antibody (Invitrogen, 0.1 μg/mL), rabbit anti-SOD2 polyclonal antibody (Invitrogen, 1:500), rabbit polyclonal anti-IL-1β antibody (Abcam, 1:500), and rabbit polyclonal anti-TNFα antibody (Abcam, 1:1000). The membranes were then incubated with an anti-rabbit horseradish peroxidase-conjugated secondary antibody (Sigma–Aldrich, 1:5000). To verify equal protein loading per line, a mouse monoclonal HRP-conjugated anti-β-actin antibody (Abcam, 1:25000) was used as an internal control. The immunoblot signals were visualized using an enhanced chemiluminescence (ECL) kit (Santa Cruz) and analyzed using ImageJ software.

The original scans of the full-length gels

Fig. S1

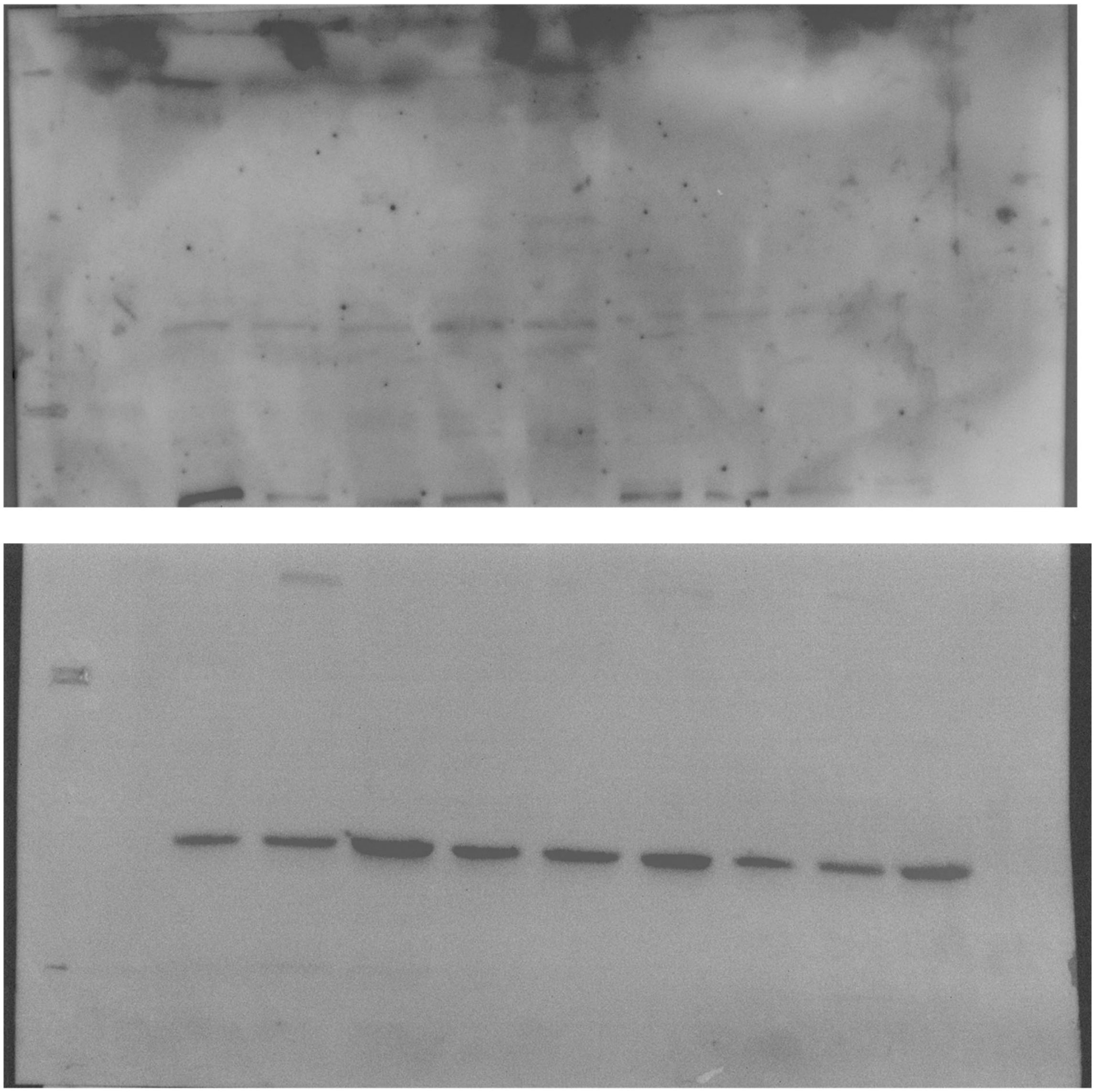

Catalase and actin (below)

Fig. S2

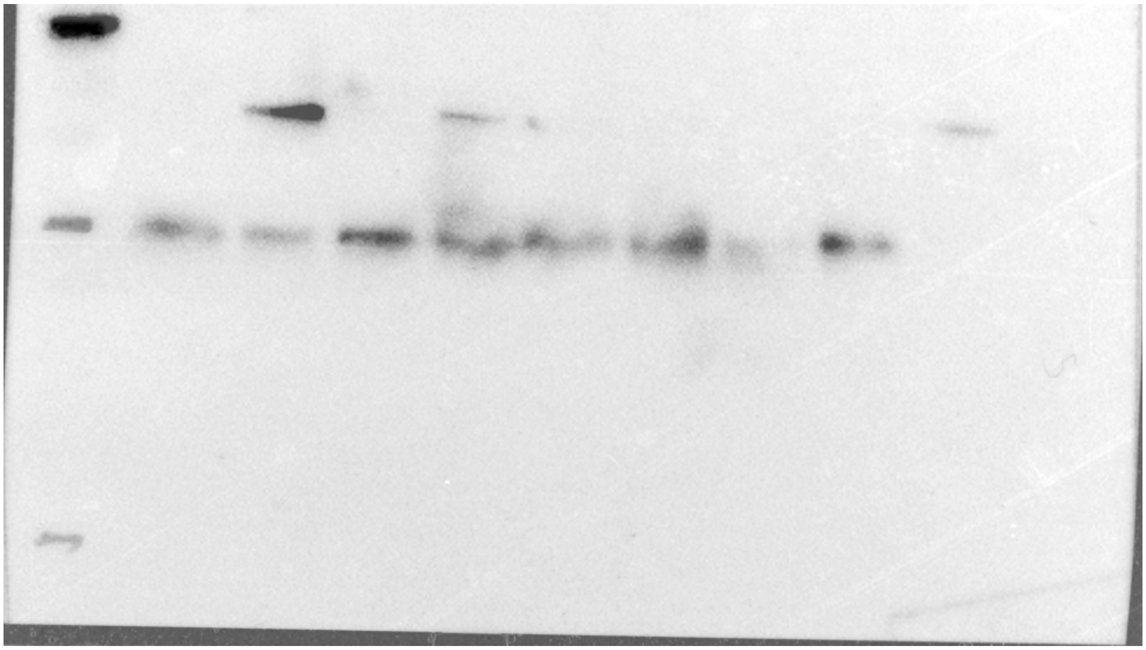

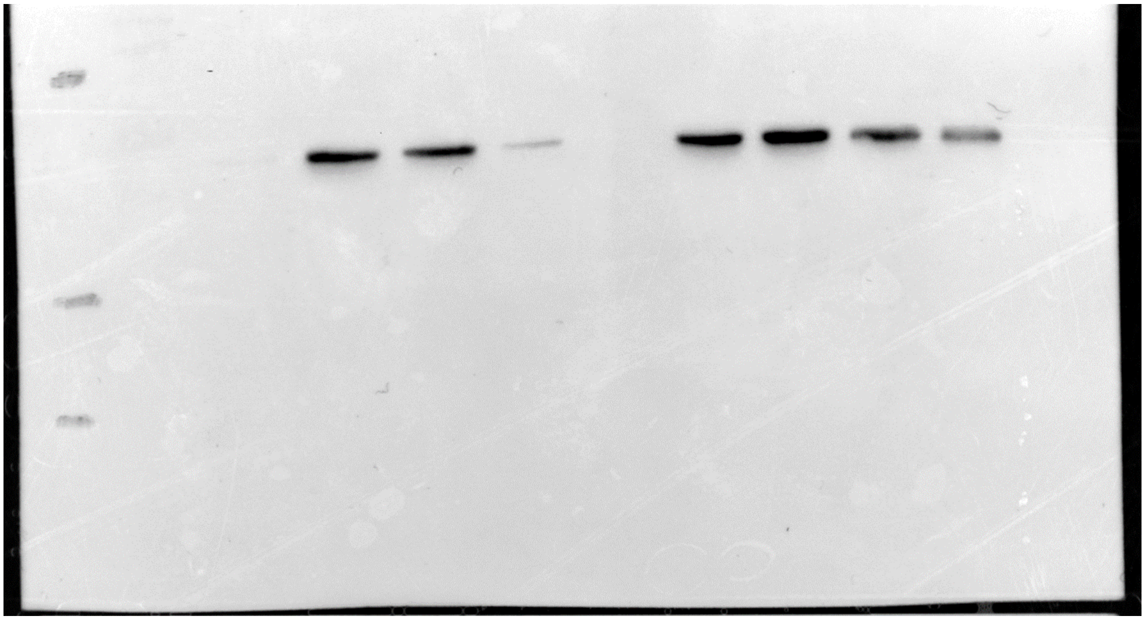

Il 1B and actin (below)

Fig. S3

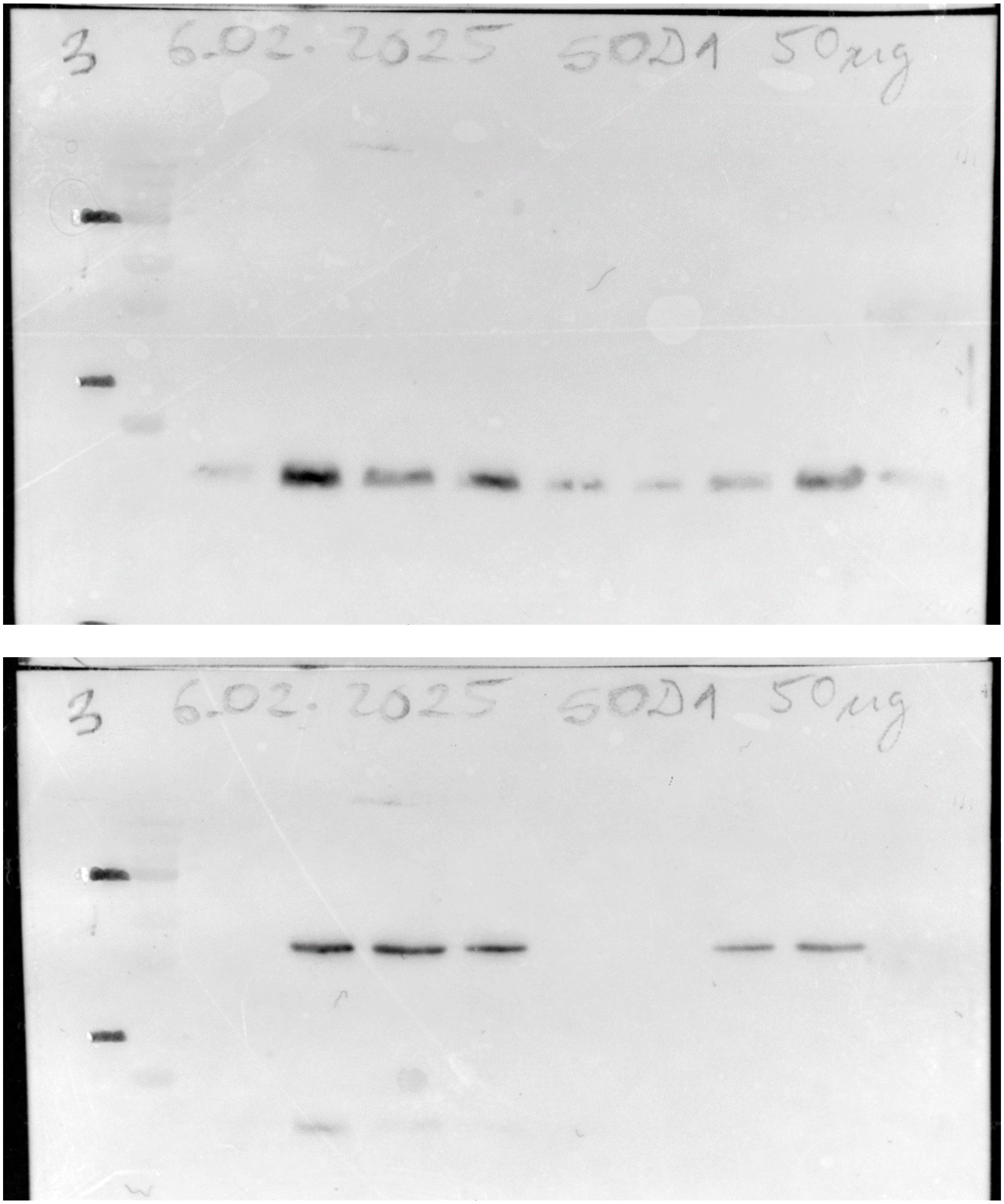

SOD1 and actin (below)

Fig. S4

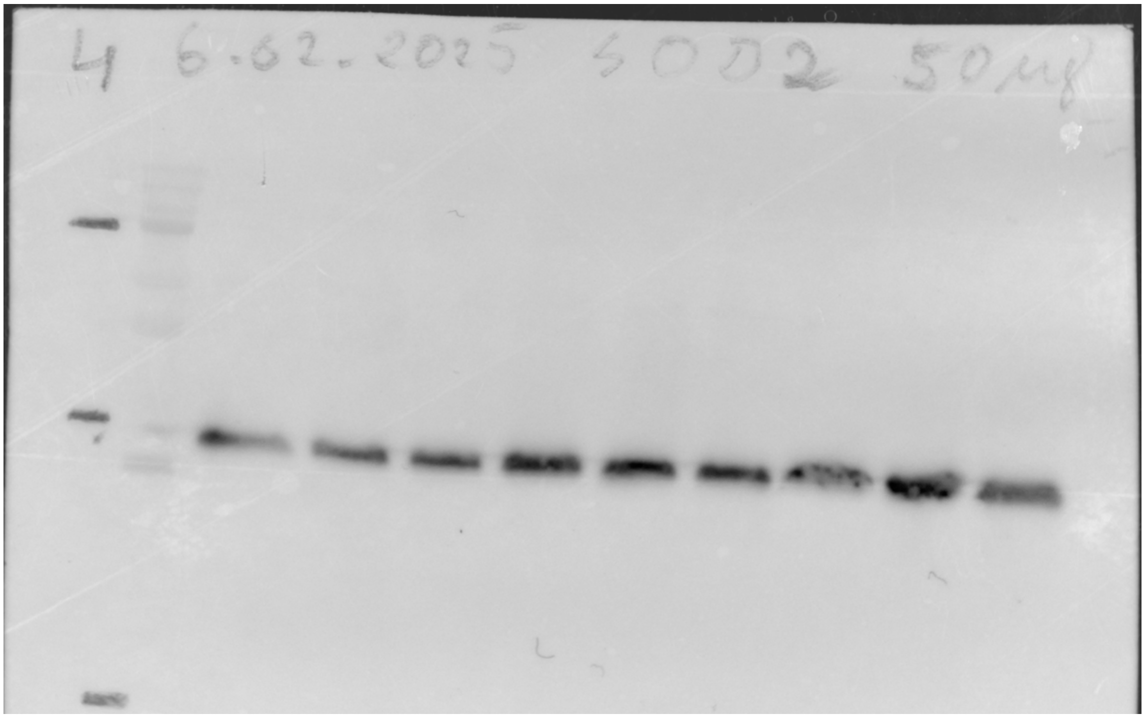

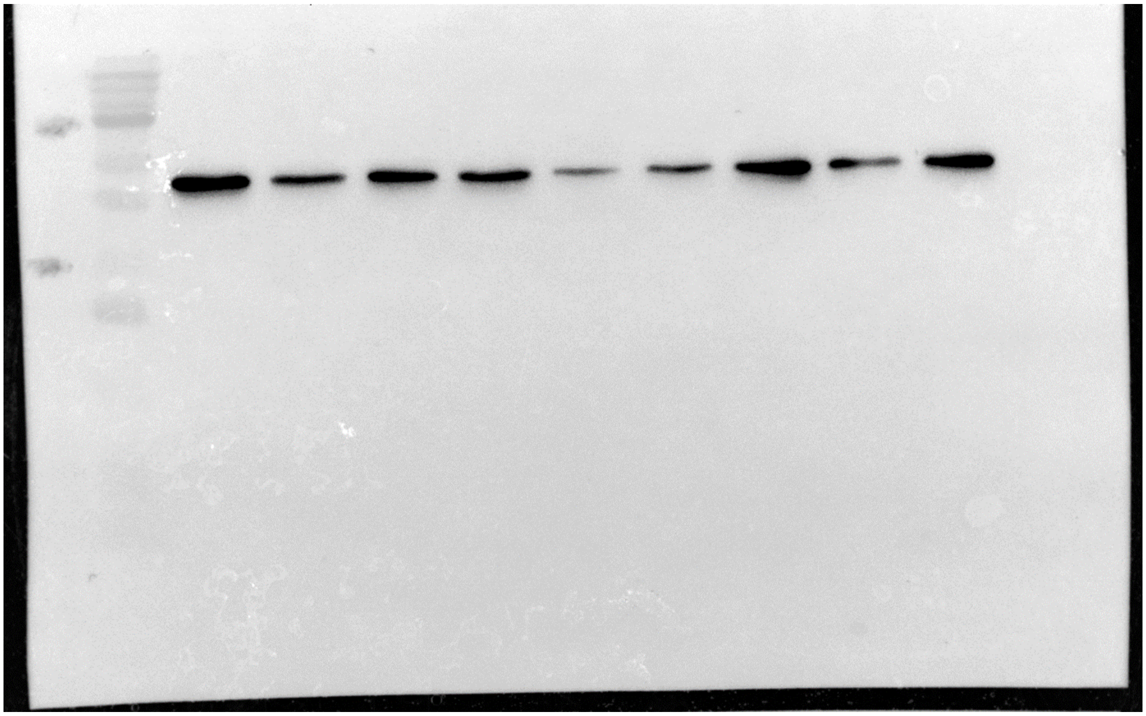

SOD2 and actin (below)

Fig. S5

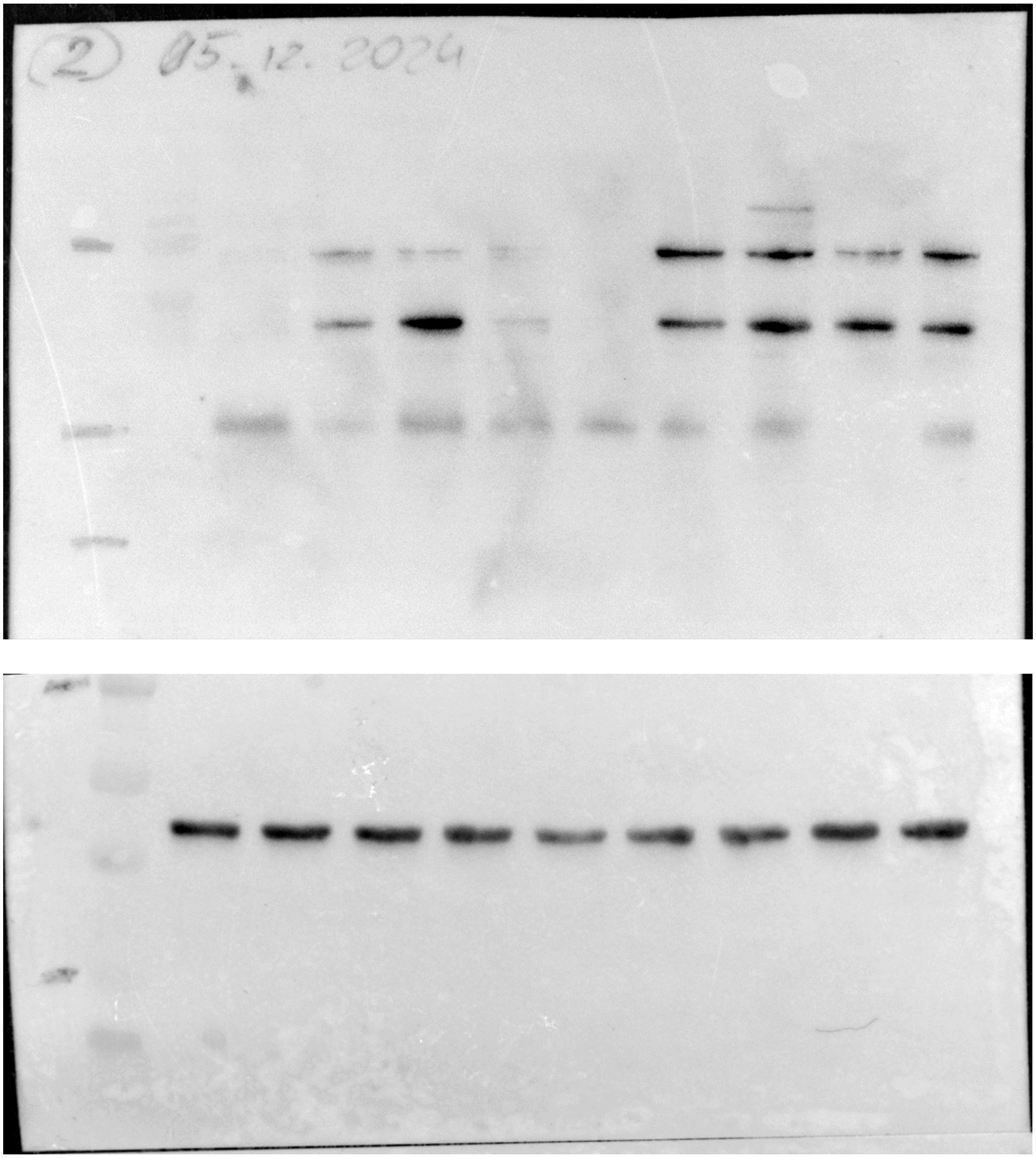

TNFα and actin (below)

Measurements of oxidative stress markers

MDA assay

The level of lipid peroxidation in the tissue samples was determined using an MDA assay kit (Merck). Approximately 10 mg of tissue was homogenized in MDA lysis buffer containing butylated hydroxytoluene (BHT). The homogenized samples were then centrifuged at 13,000 × *g* for 10 min to remove insoluble debris. After centrifugation, 200 µL of the supernatant was transferred into microcentrifuge tubes. To form the MDA-thiobarbituric acid (TBA) adduct, 600 µL of TBA solution was added to each sample and standard vial. The reaction mixture was incubated at 95°C for 60 min to ensure proper adduct formation. After incubation, the samples were cooled to room temperature in an ice bath for 10 min. For analysis, 200 µL of each reaction mixture was transferred to a 96-well plate, and the absorbance at 532 nm was measured. Background values were subtracted from all readings. The slope of the standard curve was used to calculate the MDA concentration (nM). The results are expressed as a percentage of the control value.

Protein carbonyl group content

The protein carbonyl content assay (Merck) was performed by dissolving samples in purified water and centrifuging them at 13,000 × *g* for 5 min to remove insoluble material. The samples were treated with 10% streptozocin to prevent interference, and then DNPH solution was added. The mixture was incubated at room temperature for 10 min. Protein precipitation was performed by adding 87% TCA solution, incubating for 5 min, and centrifuging at 13,000 × *g* for 2 min. The supernatant was carefully removed, and the pellet was washed with acetone, sonicated, incubated at –20°C for 5 min, and centrifuged at 13,000 × g for 2 min after each wash. After removal of the acetone, the pellet was redissolved in a 6 M guanidine solution. Each sample was transferred to a 96-well microplate, and the absorbance was measured at 375 nm. Protein concentrations were determined using the Thermo Scientific™ Pierce™ BCA protein method, and the carbonyl content was calculated as nM/mg protein. The results are expressed as a percentage of the control samples.

Total antioxidant capacity (TAC)

The collected tissue homogenates were rinsed with phosphate-buffered saline (PBS) to remove excess blood, then ground and homogenized in assay buffer (5 mM potassium phosphate, pH 7.4, containing 0.9% sodium chloride) for the antioxidant assay (Cayman Chemical). The homogenate was centrifuged at 10,000 × *g* for 10 min at 4°C, and the supernatant was collected. The samples were diluted with assay buffer prior to testing to ensure that the antioxidant levels were within the range of the standard curve. For the assay, 10 μL of the tissue homogenate was mixed with 10 μL of metmyoglobin and 150 μL of chromogen in a 96-well plate, and then 40 μL of hydrogen peroxide working solution was added to initiate the reaction. The plate was incubated on an orbital shaker for 5 min at room temperature, after which the absorbance was measured at 750 nm using a plate reader. The antioxidant capacity was quantified by comparing the absorbance quenching with a Trolox standard curve and expressed as millimolar Trolox equivalents, which were calculated as a relative percentage of the control sample.

Glutathione (GSH) measurement

The tissue was homogenized in cold buffer containing 50 mM phosphate (pH 7) with 1 mM EDTA. The homogenate was then centrifuged at 10,000 × g for 15 min at 4°C. The obtained supernatant was used for further analysis. The glutathione concentration was measured using a glutathione (GSH) assay kit (Merck) according to the manufacturer's instructions. The assay is based on the 5,5'-dithiobis(2-nitrobenzoic acid) (DTNB) method. DTNB reacts with reduced glutathione to form a yellow product, the absorbance of which is measured at 412 nm. The tissue samples (120 μL) were mixed with 120 μL of reagent A. Then, 200 μL of the sample mixture was transferred to a 96-well plate, followed by the addition of 100 μL of reagent B. The plate was incubated at room temperature for 25 min, and the absorbance was measured at 412 nm using a spectrophotometric plate reader. The GSH concentration was calculated after

subtraction of the background, and the values were compared to those of a calibrator. The final result is expressed as a percentage of the control samples.

Catalase (CAT) activity

The tissue samples were homogenized in ice-cold assay buffer. The homogenates were then centrifuged at 10,000 × *g* for 15 min at 4°C, and the supernatant was collected for the enzymatic reaction. The protein concentration was determined using the BCA assay to normalize the catalase activity measurements. Catalase activity was assessed using the decomposition of hydrogen peroxide using a Catalase Colorimetric Activity Kit (Thermo Fisher). Briefly, 25 µL of tissue lysate was mixed with 25 µL of H_2O_2. The mixture was incubated at room temperature for 30 min, after which the substrate and horseradish peroxidase were added, and the mixture was further incubated for 15 min. The absorbance was measured at 560 nm. The catalase activity was calculated as a percentage of that of the control.

Superoxide dismutase (SOD) activity

The tissue samples were homogenized using a mechanical homogenizer in ice-cold lysis buffer containing 0.1 M Trizma®-HCl, pH 7.4, 0.5% Triton™ X-100, 5 mM mercaptoethanol, and protease inhibitors. The lysates were then centrifuged at 14,000 × *g* for 5 min at 4°C, and the supernatants were collected for subsequent analysis.

SOD activity was measured using the Superoxide Dismutase (SOD) Activity Assay Kit (Merck). SOD activity is assessed by monitoring the reduction in superoxide anions. Briefly, prepared tissue lysates were mixed with reaction buffer (WST). The enzymatic reaction was initiated by the addition of xanthine oxidase, and the absorbance was measured at 450 nm using a microplate reader. The total SOD activity was calculated on the basis of the inhibition rate and expressed as a percentage of that of the control samples. The protein concentration in the lysates was determined using the BCA method, and all measurements were normalized to the same protein content.
